# The effect of a biphasic injectable bone substitute on the interface strength in a rabbit knee prosthesis model

**DOI:** 10.1186/1749-799X-8-25

**Published:** 2013-07-31

**Authors:** Vasilis Zampelis, Magnus Tägil, Lars Lidgren, Hanna Isaksson, Isam Atroshi, Jian-Sheng Wang

**Affiliations:** 1 Department of Orthopedics, Clinical Sciences, Lund University, Lund SE-221 85, Sweden; 2 Department of Orthopedics, Hässleholm Hospital, Hässleholm SE-281 25, Sweden; 3 Division of Solid Mechanics, Lund University, Lund SE-221 00, Sweden; 4 Department of Applied Physics, University of Eastern Finland, Kuopio FI-70211, Finland

**Keywords:** Injectable bone substitute, Hydroxyapatite, Calcium sulfate, Interface strength, Histomorphometry

## Abstract

**Background:**

In joint prosthetic surgery, various methods are used to provide implant stability. We used an injectable bone substitute, composed of calcium sulfate/hydroxyapatite, as bone defect filler to stabilize a tibia prosthesis in an experimental rabbit model. The aim of the study was to investigate and compare the stability of prosthetic fixation with and without the use of an injectable bone substitute.

**Methods:**

Sixteen rabbits were used and the tibia prostheses were implanted bilaterally, one side with the prosthesis alone and the other side with the prosthesis and calcium sulfate/hydroxyapatite (Cerament™). The rabbits were randomly divided into two groups and euthanized after 6 and 12 weeks, respectively. The prosthesis was extracted measuring the pull-out force in an Instron tester, and the bone surrounding the former prosthesis site was analyzed by histology, histomorphometry, and micro-computed tomography.

**Results:**

At 6 weeks no difference in maximum pull-out force was found between the prostheses fixed with or without Cerament™. At 12 weeks the maximum pull-out force for the prostheses with Cerament™ was significantly higher than that for the prostheses without Cerament™ (*p* = 0.04). The maximum pull-out force at 12 weeks was significantly higher than that at 6 weeks for the prostheses fixed with Cerament™ (*p* = 0.03) but not for the prostheses without.

**Conclusion:**

We conclude that early prosthesis-bone interface strength is not influenced by a bone substitute. However, during remodeling, the bone substitute might provide improved mechanical support for the prosthesis. The results support further studies of the use of injectable calcium sulfate/hydroxyapatite in fixation of prosthetic joint implants.

## Introduction

The gold standard for restoring bone defects is still considered to be autologous bone graft. Limitations exist, especially regarding donor site morbidity and a limited supply. In primary and revision arthroplasties, alternatives include allografts, bone cement, or the use of long-stemmed prostheses [1,2]. By using allograft bone, a potential risk for blood-borne diseases such as hepatitis and HIV is introduced. Alternatives have been tried using artificial bone grafts in the form of pellets, alone or mixed with a bone graft [3]. Several types of injectable bone graft substitutes have been developed to fill bone defects in fractures and osteotomies [4]. The choice and composition of the artificial bone graft material determine the mechanical properties of the bone substitute, not only immediately after implantation but also during the whole remodeling period. If the material is resorbed too rapidly, the strength might be hampered with insufficient bone support for the prosthesis. If the material is remodeling too slowly, living new bone will not be able to replace the bone substitute in order to secure long-term fixation. Thus, a properly chosen bone substitute or graft should be used to reconstruct bone defects in order to provide stability and support during the whole remodeling period. The selection of an appropriate bone graft is influenced by the size of the bone defect, the biological prerequisites of the bone graft site, and whether the graft is required for structural support.

We hypothesized that an injectable bone substitute can be used to fill the gap between the bone and the prosthesis and that combining a slowly and a fast resorbing material into one can utilize the benefits of the properties of the two materials, providing improved fixation of a prosthesis. In the present study, we used injectable calcium sulfate/hydroxyapatite bone substitute [5,6] containing (1) a fast-resorbing calcium sulfate, allowing for a fast replacement of the bone substitute with new-forming bone [7], and (2) a hydroxyapatite component acting as a long-term osteoconductive matrix embedded into the forming bone tissue [8,9], providing mechanical support for the prosthesis during remodeling. We evaluate this biphasic material in a primary tibia prosthesis model in rabbits, a model previously used to study morsellized bone graft [10]. The aim of the study was to investigate and compare the stability of prosthetic fixation with and without the use of an injectable bone substitute in an experimental rabbit model.

## Materials and methods

### Animals

Sixteen 6-month-old New Zealand white rabbits were used. Twelve of the 16 rabbits were used for the mechanical testing and histological analysis, while 4 rabbits were used for computed tomography (CT) analysis. The rabbits were skeletally mature (weight 4.0 to 4.5 kg) and had free access to food and water. The animals arrived at the laboratory 1 week prior to the operation to acclimatize. All animal procedures were carried out according to the institutional guidelines and were approved by the ethic animal research committee (M134-10) at the Skåne University Hospital, Lund, Sweden.

### Bone substitute material

The injectable bone substitute Cerament™ (Bone Support AB, Lund, Sweden) was used consisting of 60% calcium sulfate and 40% hydroxyapatite (HA) [5,11]. The ratio results in a mixture with compressive strength (wet) of 5–8 MPa, comparable to trabecular bone. The HA is of medical grade and tested according to American standard (ASTM F1185). The bone substitute powder is mixed with iohexol (180 mg I/ml), a low-osmolarity nonionic iodinated contrast agent to form an injectable paste. The working time and injectability of the substance is 6 min using a 16G cannula, and the final setting time is 15 to 20 min. The maximum setting temperature is 38°C.

### Surgical protocol

**Figure 1:**
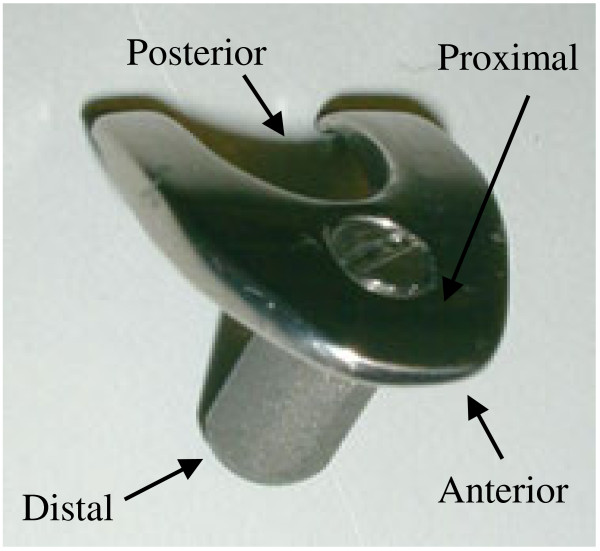
**The rabbit tibia prosthesis.** The prosthesis stem is made of titanium, has a 3.5-mm diameter and 8-mm length, and is not side specific. The tibia tray can be removed and replaced by a hook for mechanical pull-out test.

A rabbit tibia prosthesis has been designed for this model [10]. It is manufactured in one size and identical for left and right knees (manufactured by the Dept. of Medical Technique in Lund University Hospital, Lund, Sweden). The stem is round, 3.5 mm in diameter, and 8 mm long. A tibia plate, mimicking the tibia plateau shape, was mounted on the top of the stem and fixed with a screw (Figure 1). A rubber plug (4 mm in diameter) was placed distally to the prosthesis to prevent material leaking into the distal part of the bone marrow cavity. Prior to surgery, a protocol was made of random insertion of the prostheses with or without bone substitute in the left and right tibiae. For each time point (6 and 12 weeks), six rabbits were used respectively, and the sample size was based on the results and standard deviations of previous studies using this model [10,12].

The rabbits were anesthetized with subcutaneous Hypnorm® (fentanyl/fluanisone, 0.3 ml/kg) and intramuscular Stesolid® (diazepam, 2.5 mg/kg). All rabbits received a single intramuscular dose of 0.4 ml Streptocillin® (hydrostreptomycin and benzylpenicillin) 10 min before operation. Prior to surgery, the skin was shaved and washed with iodine. Both tibiae were operated on. Under aseptic conditions, a midline skin incision was made over the knee joint and the joint was approached through a medial parapatellar incision. The patella was mobilized laterally, the menisci removed, and a 1.5-mm area of the tibia articular surface resected with an electric saw. The bone marrow cavity was opened with an awl and reamed with a 3.6-mm drill. Cerament™ bone graft substitute was used for fixation of the tibia prosthesis. Cerament™ powder (200 mg) was mixed with 87 μl of iohexol, which in turn generated a liquid and powder ratio of 0.43 ml/g. The material was mixed for 3 min in a Petri dish and inserted into the tibia cavity. The prosthesis was gently inserted in the tibia marrow cavity by digital pressure and thereafter gently tapped into the bone using a mallet (Additional file 1). In the contralateral control side, the bone marrow cavity was simply washed with saline after reaming and the prosthesis implanted. Joint stability and range of motion were controlled, and the joint capsule and the subcutaneous tissues were closed in two separate layers. After surgery, the animals were allowed to move freely in their cages. The animals were randomly divided into two groups to be sacrificed 6 or 12 weeks after prosthetic implantation. The animals were sacrificed by an overdose of pentobarbital, and six specimens in each of the 6-week and 12-week groups were randomly selected for a pull-out test and two specimens for a micro-CT analysis. The specimens from the pull-out tests were subsequently fixed in formalin for histology and histomorphometric assessment.

### Pull-out test

The tibias with the prosthesis implant were cleansed from soft tissue, and the distal parts were fixed with bone cement in a plastic tube and mounted on an Instron 8511 load frame with an MTS TestStar II controller (Canton, MA, USA). The prosthetic tibia plate was unscrewed and replaced by a hook screwed onto the stem (Additional file 2). The implant was first preloaded up to 1 N, and thereafter, the pull-out test was performed under displacement control at 2 mm/min, with the tester being unaware of group belonging of the specimen. The peak force was recorded and the data from the pull-out test were compared for the prostheses with and without Cerament™ at 6- and 12-week time points, respectively.

### Histological and histomorphometric analyses

After the pull-out test, the same specimens were prepared for histological analysis. The specimens were decalcified (10% EDTA), embedded in paraffin, cut into 5-μm sections, and stained with hematoxylin and eosin. The specimens were cut longitudinally in 300-μm intervals, and five sections were obtained from each specimen. The surface area of the material remaining in the bone defect as well as the bone integration measured as the percentage of the interface and ingrowth was analyzed using a light microscope (Olympus Bx50, Shinjuku-ku, Japan) and an image analysis system (Aperio Image Scope v.11.12-752, Vista, CA, USA). The newly formed bone was marked in the histology images, and the total bone interface length was measured. The percentage of bone contact for each specimen in all five sections was calculated. The areas on the bottom part of the prosthesis were not included in the measuring process since tissue had detached in some specimens during the pull-out test. The percentage of bone contact was calculated as the bone contact length divided by the total prosthesis length.

### Micro-computed tomography

**Figure 2:**
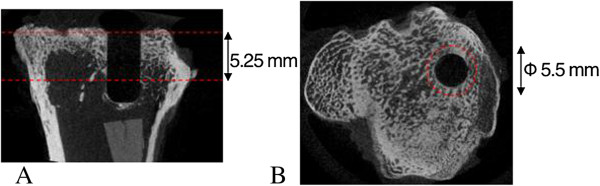
**Micro-CT regions of interest. (A)** 5.25 mm was analyzed by drawing a **(B)** 5.5-mm circular region of interest. Before calculating the BV/TV, the volume of the prosthesis was subtracted.

Four of the rabbits, two from each period (6 and 12 weeks), were evaluated with micro-CT. The proximal tibiae were scanned with an isotropic voxel size of 35 μm (SkyScan 1172, v. 1.5, SkyScan, Aartselaar, Belgium) at the energy settings of 100 kV and 100 μA, using an aluminum filter of 0.5 mm, and ten repeated scans. The images were reconstructed using NRecon (v 1.5.1.4, SkyScan, Aartselaar, Belgium) by correcting for ring artifacts and beam hardening. Calibration of bone mineral density (BMD) was carried out according to the system manufacturer's protocol by scanning of a water phantom and two hydroxyapatite phantoms of known density (0.25 and 0.75 g/cm^3^). Mineralized bone tissue was assumed to have a BMD over 0.60 g/cm^3^, resulting in gray-scale values of 88–255. A circular region of interest was defined as the 1-mm outline of the circumference of the prosthesis. Thus, a region with a total of 5.5 mm in diameter was analyzed in which the 3.5 mm of the prosthesis cavity area was included. The length of the prosthesis was analyzed for 5.25 mm (150 images, Figure 2). Bone volume fraction (BV/TV) was calculated after subtracting the volume of the implant from the region of interest, using CTAn (v.1.9.1.0 SkyScan, Aartselaar, Belgium).

### Statistical analysis

We have used a sample size of 12 rabbits previously in studies of morsellized impacted bone grafts studying the impact of mechanical load on the graft [10] as well as using bone morphogenic protein (BMP) in addition [12]. With a similar SD and the same clinically significant difference, we anticipated that we would be able to reach significance with similar differences and with the same power. The additional four rabbits were used for the CT analysis.

The SPSS® Statistics 20 (IBM®, Chicago, IL, USA) was used for statistical analyses. The Wilcoxon signed-rank test was used for paired comparison between right and left sides and the Mann Whitney *U* test for comparing groups at 6 and 12 weeks. Results are presented as median (interquartile range (IQR)).

## Results

All rabbits completed the study period. Food intake and weight bearing gradually normalized in all animals. One rabbit sustained a patellar subluxation 6 weeks after surgery but ambulated normally and was therefore included. One rabbit showed signs of a superficial wound infection. Infection was present in the side where the prosthesis was fixed without Cerament™ at the 12-week group. At the pull-out test, the prosthesis in the infected side was loose, whereas in the contralateral side, in which Cerament™ was used, the pull-out force was 227 N. In the test protocol, infection, fracture, and luxation were defined as a cause for exclusion. Therefore, the animal was excluded from the study.

### Pull-out test

After 6 weeks, no differences in maximum pull-out force were found between the prostheses fixed with or without Cerament™ (*p* = 0.92). However, at 12 weeks the maximum pull-out force for the prostheses fixed with Cerament™ was significantly higher than that for the prostheses without Cerament™ (*p* = 0.04). The maximum pull-out force increased between 6 and 12 weeks in the prostheses fixed with Cerament™ (156 to 256 N; *p* = 0.03) but showed no significant difference in the prosthesis fixed without Cerament™ (143 to 175 N; *p* = 0.20) (Table 1).

**Table 1:** Comparison of maximum pull-out force for the prostheses fixed with and without Cerament™

	**Maximum pull-out force (N)**		***p *value**
	**6 weeks**	**12 weeks**	
	**(*N *= 6)**	**(*N *= 5)**	
	**Median (IQR)**^**a**^	**Median (IQR)**^**a**^	
Prostheses without Cerament™	143 (78–179)	175 (115–230)	0.20
Prostheses with Cerament™	156 (99–210)	234 (195–243)	0.03
*p* value	0.92	0.04	

^a^Interquartile range is equal to the difference between the upper and lower quartiles.

### Histological and histomorphometric findings

**Figure 3:**
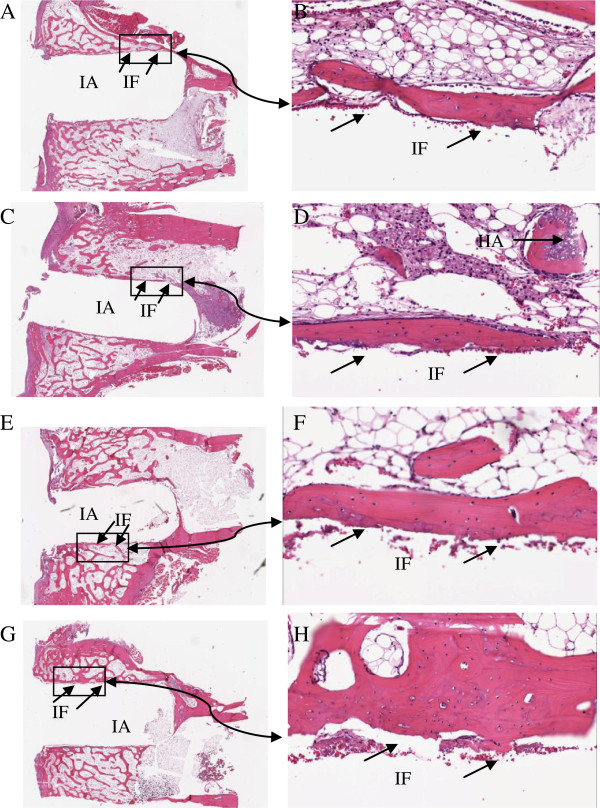
**Histology slides of the bone interface.** Histology slides of the bone interface in the prosthesis fixed without Cerament™ **(A**, **B)** and with Cerament™ **(C**, **D)** at week 6, and in the prosthesis fixed without Cerament™ **(E**, **F)** and with Cerament™ **(G**, **H)** at week 12 (original magnification ×1 and ×20, respectively). *IA*, implant position before removal; *IF*, interface area between the graft and the host; *HA*, hydroxyapatite particles integrated with new-formed trabecular bone.

**Figure 4:**
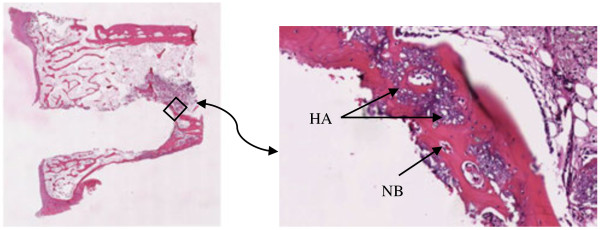
**Histology slides showing the bone integration.** Histology slides showing the bone integration at week 6 in the prosthesis fixed with Cerament™ (original magnification ×1 (*left*) and ×20 (*right*)) in the distal part of the prosthesis. Hydroxyapatite particles surrounded by new-formed bone. *NB*, new bone; *HA*, hydroxyapatite particles.

The histology after 6 and 12 weeks showed new-formed bone in both groups. However, differences in bone ir-regularity and thickness were noticed. In the 6-week group, the arrangement of the bone matrix appeared slightly disrupted and the surface of the new bone at the interface toward the prosthesis was coarse. In the 12-week group, the new-formed bone was smoother and the bone matrix around the prostheses was more regularly arranged, i.e., more mature (Figure 3). Over weeks, the calcium sulfate component appeared to have been replaced by new-formed trabecular bone, which also integrated with the hydroxyapatite component (Figure 4). Histology in the rabbit excluded from the study 12 weeks after surgery showed signs of fibrous tissue in the side where the prosthesis was fixed without Cerament™ and infection was present, and new-formed trabecular bone which integrated with the hydroxyapatite component in the side where Cerament™ was used.

The bone contact was between 60% and 66% both at 6 and 12 weeks independent of the fixation method of the prosthesis. Hence, the bone contact did not differ between the groups with prostheses fixed with or without Cerament™ at either time point.

### Micro-computed tomography

**Figure 5:**
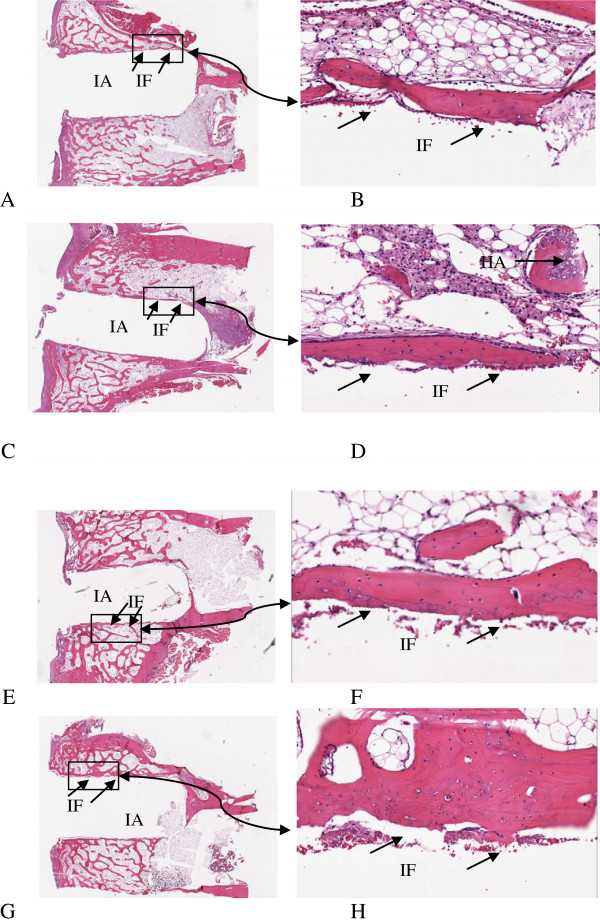
**Micro-CT images.** Micro-CT 3D rendering of the proximal tibia **(A)**, and the bone within a 5.5-mm-diameter region **(B**, **C)** showing high bone contact with smooth surface in both groups both after 6 and 12 weeks. BV/TV ranged from 35% to 44% in the region of interest.

The bone contact was high and the surface was smooth in both groups both after 6 and 12 weeks (Figure 5). The BV/TV ranged from 35% to 44% in the region of interest.

## Discussion

In joint arthroplasty, the role of the bone graft or bone substitute is different than that in many other situations like in fracture healing or nonunion treatment. Firstly and most importantly, the graft should provide mechanical support for the prosthesis, not only during the immediate postoperative period but also during the later remodeling period. Secondly, it is desirable that the prosthesis-bone graft interface consists of living bone, capable of a constant repair due to the accumulated stress in the supporting bone during loading. Particulate bone grafts are used to fill voids in primary and revision arthroplasties. It has been speculated that the reason for the good long-term results achieved by the use of morsellized impacted bone graft in revision surgery lies in it being remodeled more slowly or maybe to a lesser extent [13,14]. In both animal models [15] as well as in humans [16], BMP has been tried to enhance remodeling with a less than acceptable result. We know today that although BMP induces bone formation, it also induces bone resorption through the RANKL system [17]. The resorption can be delayed by using an anti-resorptive drug like a bisphosphonate [12], which by speculation also would lead to improved bone quality also in humans [18].

The advantage of using an injectable bone substitute encompasses the ability to fill out the entire space between the prosthesis and the bone, thereby adding to the primary stability of the prosthesis. In contrast to PMMA, the bone substitute is bone conductive and the bone defect is at least theoretically replaced by host bone. The important factor for the stability during remodeling is not only the strength of the material at insertion but rather the speed of resorption and thereby the strength over time.

In the present study, we used a bone substitute composed of calcium sulfate and hydroxyapatite, which provides stabilization not only initially but also later during the period of remodeling and biological stabilization. We use a primary surgery model and not a revision which is a limitation of the study and which might influence the magnitude of pull-out strength. Biologically, the fast resorption of the calcium sulfate is the key feature for later bone remodeling. The resorption creates space for the ingrowing bone, which in turn is able to integrate with the nonresorbing hydroxyapatite particles, and hypothetically, the bone-HA composite strength thereby increases at the interface. The biphasic Cerament™ was used in a recent study of 33 patients who underwent percutaneous vertebroplasty after osteoporotic and/or traumatic vertebral fractures. Radiological and clinical outcome was assessed by radiography, CT, and MRI, and 12 months after surgery, the fractures were stabile and new bone formation visible [19].

In our present animal study, the pull-out force of the prostheses fixed with Cerament™ increased significantly between 6 and 12 weeks. At 12 weeks the maximum pull-out force for the prostheses fixed with Cerament™ was significantly higher than that for the prostheses without Cerament™. A biological reinforcement of the fixation by a gradual substitution of the bone substitute with living bone could be important for long-term fixation. As shown in both histology and micro-CT, the proportion of living bone at the interface at both 6 and 12 weeks was similar in the two groups, suggesting a qualitative difference rather than a quantitative one. Most importantly, no adverse effects were found by the bone substitute. We believe that Cerament™ can be used as bone void filler also in prosthetic fixation; however, further studies are required.

## Competing interests

LL owns stocks in the company providing the bone substitute. The other authors declare that they have no competing interests.

## Authors’ contributions

VZ, J-SW, and HI validated and analyzed the data. MT and J-SW conceived and developed the study design. VZ, J-SW, MT, IA, LL, and HI all discussed the analysis and participated in writing the manuscript. All authors read and approved the final manuscript.

## Supplementary Material

Additional file 1 **The surgical approach and the prosthesis fixation. (A)** Resection of the tibia plate. **(B)** Preparation of the tibia medullary canal. **(C)** Cerament™ fixation. **(D)** The fixation of the prosthesis on the tibia plate. Click here for file

Additional file 2 **Pull-out test.** The fixed tibia was mounted on an Instron 8511 load frame with an MTS TestStar II controller. Click here for file
