# Supplementary figures and images for: The effect of a biphasic injectable bone substitute on the interface strength in a rabbit knee prosthesis model
Source: J Orthop Surg Res. 2013 Jul 31;8:25. doi: 10.1186/1749-799X-8-25 (PMC3734137; doi:10.1186/1749-799X-8-25)

Figure 2


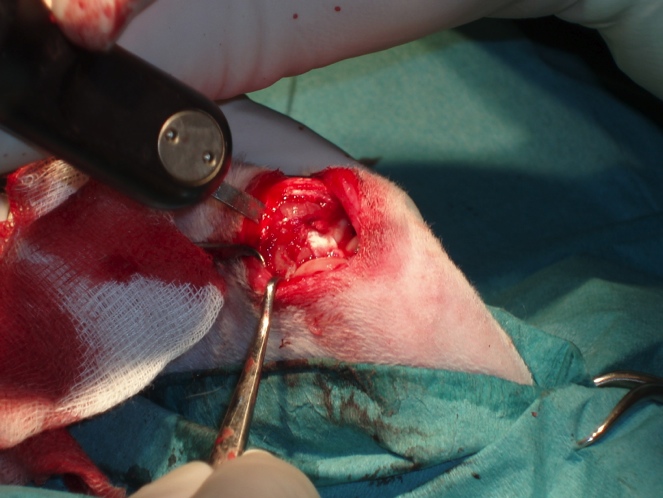

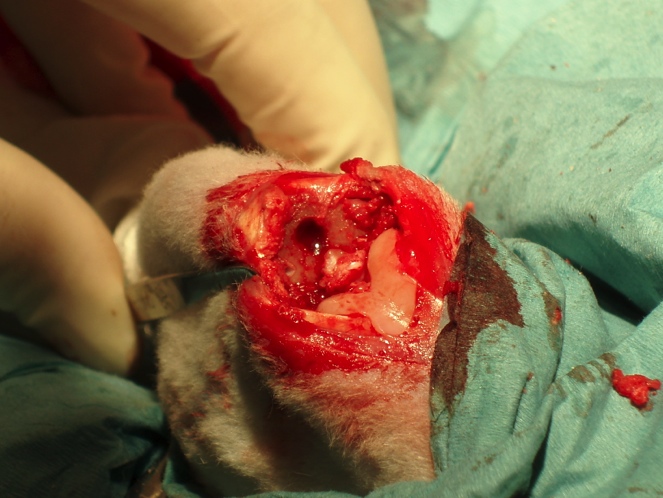


A B


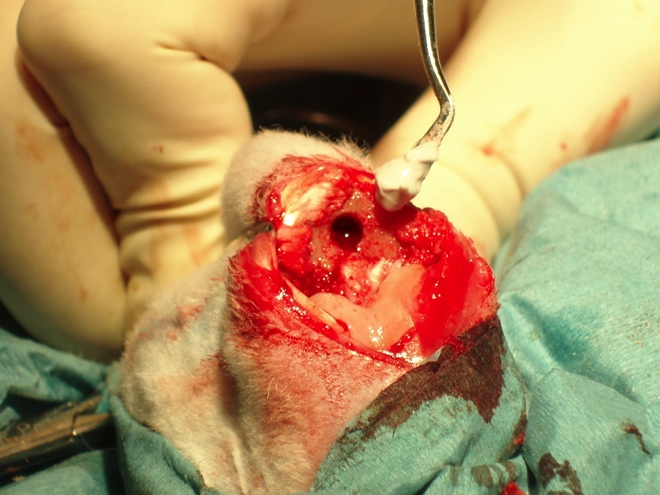

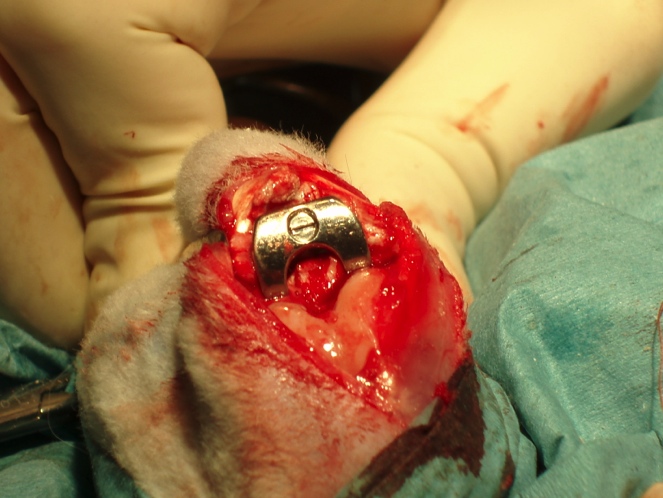


C D

Supplement: Additional file 1 — The surgical approach and the prosthesis fixation. (A) Resection of the tibia plate. (B) Preparation of the tibia medullary canal. (C) Cerament™ fixation. (D) The fixation of the prosthesis on the tibia plate. [file 1749-799X-8-25-S1.doc]

Figure 3.


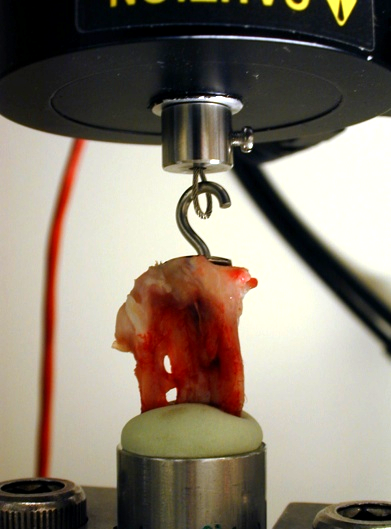

Supplement: Additional file 2 — Pull-out test. The fixed tibia was mounted on an Instron 8511 load frame with an MTS TestStar II controller. [file 1749-799X-8-25-S2.doc]
